# An Audit on the Adherence to Antipsychotic Prescription Policy for the Management of Delirium in the Medical Wards

**DOI:** 10.1192/bjo.2022.490

**Published:** 2022-06-20

**Authors:** Manjula Simiyon, Jiann Loo, Catherine Baker, Peter Lepping, Steven Jones

**Affiliations:** 1 Betsi Cadwaladr University Health Board, Wrexham, United Kingdom; 2 University of Chester, Chester, United Kingdom

## Abstract

**Aims:**

This audit aimed to assess the adherence to the anti-psychotic policy for delirium in the medical wards. It aimed to assess compliance with each of the guidelines mentioned in the health board's policy which is based on the National Institute for Health and Cares Excellence (NICE) guidelines.

**Methods:**

After registering the audit, the Acute medical ward was approached for the hospital numbers of all the patients admitted in the months between January and March 2021, and 70 case records were screened. Case notes of patients above 18 years who were diagnosed with delirium including those after managing alcohol withdrawal were included. Those who were admitted only with alcohol withdrawal delirium were excluded. 47 case records were selected for data collection. A proforma was prepared based on the policy available in the intranet and data were entered.

**Results:**

Retrospective data of 47 patients who had delirium were analysed which included 18 males and 29 females. The mean age of the participants was 80.7 years (range 40–101; SD + 30). The mean days of referral after admission were 28(+7.07). 34%were diagnosed to have delirium by the treating team,8.5% were diagnosed by the Emergency Department (ED) team and 57.4% were diagnosed by the liaison psychiatric team. 57% had another psychiatric diagnosis. The cause for delirium was mentioned in 55% of the records and the most common cause was urinary tract infection (31%) followed by multifactorial delirium (27%). Antipsychotics were prescribed for 57% and among those who received 74% received risperidone, 15% received olanzapine, and 11% haloperidol. Compliance was 100% in prescribing appropriate antipsychotics, maximum dose, investigations (expect x-ray chest and CT scan), only 54% compliance was observed with regards to stopping the antipsychotic before discharge and in 23% it was mentioned to be monitored by the GP and another 23% by the treating team.

**Conclusion:**

This audit has displayed the lacuna in the prescription of antipsychotics for patients diagnosed with delirium. Periodic programs will be planned and executed for training the liaison practitioners and the staff in the medical wards regarding the diagnosis and management of delirium especially the prescription of antipsychotics. A re-audit will be conducted after 6 months.

